# What Can Gamma Delta T Cells Contribute to an HIV Cure?

**DOI:** 10.3389/fcimb.2020.00233

**Published:** 2020-05-19

**Authors:** Jennifer A. Juno, Stephen J. Kent

**Affiliations:** ^1^Department of Microbiology and Immunology, The Peter Doherty Institute for Infection and Immunity, The University of Melbourne, Melbourne, VIC, Australia; ^2^Department of Infectious Diseases, Melbourne Sexual Health Centre, Alfred Health, Central Clinical School, Monash University, Clayton, VIC, Australia; ^3^ARC Centre of Excellence in Convergent Bio-Nano Science and Technology, The University of Melbourne, Melbourne, VIC, Australia

**Keywords:** gamma delta T cell, Vδ2, Vδ1, HIV, immunotherapy

## Abstract

Elimination of the latent HIV reservoir remains a major barrier to achieving an HIV cure. In this review, we discuss the cytolytic nature of human gamma delta T cells and highlight the emerging evidence that they can target and eliminate HIV-infected T cells. Based on observations from human clinical trials assessing gamma delta immunotherapy in oncology, we suggest key questions and research priorities for the study of these unique T cells in HIV cure research.

## Introduction

Efforts to eliminate the latent HIV reservoir have, to date, been unsuccessful, outside of aggressive chemotherapy and stem cell transplants from genetically resistant donors (Hutter et al., 2009; Gupta et al., 2020). Killing of infected cells with minimal viral replication, or killing of infected cells after latency reversal, is likely to be critical in the long-term containment or eradication of HIV. Novel approaches to clear infected cells now include the study and manipulation of highly cytotoxic lymphocyte subsets beyond traditional CD8+ CTL (Garrido et al., 2018a,b). It has become apparent from both *in vitro* studies and human clinical trials in oncology that gamma delta T (γδT) cells exhibit remarkable cytotoxicity (Simoes et al., 2018) and the potential for safe clinical use in human immunotherapy (Silva-Santos et al., 2019). A number of excellent reviews have recently described the use of human γδT cells in clinical trials for cancer treatment (Lo Presti et al., 2017; Godfrey et al., 2018; Pauza et al., 2018; Silva-Santos et al., 2019). Here, we will discuss how advances in gdT immunology have identified these cells as potential anti-HIV effectors, and what remains to be established regarding the efficacy of γδT cells as components of an HIV cure intervention.

## Human Gamma Delta T Cell Subsets

Human γδT cells are typically classified on the basis of their TCR delta chain, of which there are 8 variants (Hayday, 2000). In peripheral blood, up to 90% of γδT cells express the Vδ2 chain (Triebel et al., 1988). The majority of Vδ2 cells pair with Vγ9 and form the well-studied population of phosphoantigen-reactive γδT cells (Tanaka et al., 1995). In contrast, Vδ2-negative γδT s dominate at many mucosal sites, including the gut (Lundqvist et al., 1995). These Vδ2- γδT cells tend to express either the Vδ1 or Vδ3 chain, with a variety of Vγ chain pairings (Groh et al., 1998). Vδ1 cells typically, but not always (Hviid et al., 2000), form a minor population of the circulating γδT cell compartment.

Vδ2Vγ9 cells (herein referred to as Vδ2 cells) form a polyclonal T cell population that rapidly expands postnatally, most likely due to persistent antigen exposure or other inflammatory stimuli (Pauza and Cairo, 2015; van Der Heiden et al., 2020). The Vδ2Vγ9 TCR recognizes pyrophosphate antigens, which include isopentenyl pyrophosphate (IPP) and the potent microbial metabolite (E)-4-Hydroxy-3-methyl-but-2-enyl pyrophosphate (HMB-PP) (Triebel et al., 1988). Like other unconventional T cells, however, Vd2 cells can also respond to TCR-independent stimuli, including cytokines such as IL-12 and IL-18, and various NK cell receptor ligands (Provine et al., 2018). Interestingly, phosphoantigen-reactive γδT cells are found only in humans, non-human primates, and alpacas, with no γδT cells in mice recognizing similar antigens (Fichtner et al., 2020). Owing in part to the ease with which they can be expanded *in vitro*, Vδ2 cells have been well characterized in human health and disease, and many of their defining features have been recently reviewed (Tyler et al., 2015; Davey et al., 2018). Notably, Vδ2 cells are commonly depleted during chronic HIV infection, with incomplete restoration among ART-treated cohorts (Hinz et al., 1994; Poccia et al., 1996; Wesch et al., 1998; Enders et al., 2003; Poles et al., 2003; Li et al., 2014, 2015; Cimini et al., 2015; Strbo et al., 2016; Bhatnagar et al., 2017). We have recently reviewed the impact of HIV infection and treatment on both circulating and tissue-resident γδT cells (Juno and Eriksson, 2019).

In contrast to Vδ2 cells, the antigen specificity of Vδ1 cells remains largely unknown. The Vδ1 cell population includes cells with specificity for R-phycoerythrin and EphA2 (Willcox and Willcox, 2019), CD1c- and CD1d-restricted lipid antigens (Uldrich et al., 2013; Roy et al., 2016; Willcox and Willcox, 2019), and the antigen presenting molecule MR1 (Le Nours et al., 2019). Vδ1 phenotype and frequency is markedly altered by infections such as malaria (Hviid et al., 2019) and human cytomegalovirus (HCMV) (Pitard et al., 2008; van Der Heiden et al., 2020). HCMV seropositivity is typically associated with substantial clonal expansion within the Vδ1 population, and differentiation toward a terminally differentiated (CD27-CD45RA+) phenotype (Davey et al., 2017; van Der Heiden et al., 2020). In contrast to Vδ2 cells, the Vδ1 cell population is significantly expanded during HIV infection and ART (De Paoli et al., 1991; De Maria et al., 1992; Rossol et al., 1998; Wesch et al., 1998; Poles et al., 2003; Poggi et al., 2004; Fausther-Bovendo et al., 2008; Li et al., 2014; Cimini et al., 2015; Olson et al., 2018; Chevalier et al., 2019), the implications of which are largely unknown (Juno and Eriksson, 2019).

## Anti-HIV Activity of Gamma Delta T Cells

Efforts to eliminate the HIV reservoir following latency reversal have traditionally focused on the utility of antigen-stimulated conventional CD8+ T cells to kill HIV-infected cells (Shan et al., 2012; Deng et al., 2015). Limitations to this approach include the requirement for autologous CD8+ T cells to be collected and pre-stimulated for each individual (Shan et al., 2012), as well as a high burden of CTL escape variants in the latent reservoir (Deng et al., 2015). More recently, other lymphocyte subsets have been considered for use in HIV cure approaches. Evidence suggests that cytokine-treated NK cells can eliminate HIV-infected T cells following *ex vivo* latency reactivation, although IL-15 treatment downregulates the expression of the key NK cell receptor NKp46, which may be undesirable *in vivo* (Garrido et al., 2018a). With a transcriptional phenotype that blends characteristics of both NK and CD8+ T cells (Gutierrez-Arcelus et al., 2019; Pizzolato et al., 2019), γδT cells are intriguing candidates to mediate anti-HIV effector functions. Indeed, γδT-mediated inhibition of HIV replication has been recognized for more than 20 years (Poccia et al., 1999).

Like NK cells (Fehniger et al., 1998; Oliva et al., 1998), stimulated γδT cells can produce sufficient β-chemokines to block HIV entry into either CCR5+ or CXCR4+ CD4+ T cells (Poccia et al., 1999; Omi et al., 2014). In the context of HIV cure approaches, however, it is the potent cytolytic function of γδT cells that makes them strong candidates for immunotherapy. Early reports suggested that direct cytotoxicity toward HIV-infected cells was largely restricted to Vδ2 cell clones (Wallace et al., 1996; Poccia et al., 1997), with little to no cytotoxicity observed among Vδ1 cell lines (Wallace et al., 1996). More recently, Vδ1 recognition and killing of HIV-infected CD4+ T cells has been demonstrated (Fausther-Bovendo et al., 2008). Although it is challenging to determine the extent to which γδT cells contribute to natural control of HIV infection in cross-sectional studies, elite/viral controllers do exhibit higher frequencies of Vδ2 cells than untreated or antiretroviral treated normal progressors (Riedel et al., 2009; Chevalier et al., 2019). A study in non-human primates identified a relationship between cervical Vδ2 frequency and simian immunodeficiency virus (SIV) viral load (Tuero et al., 2016), which supports the possibility of a protective role for these cells during infection.

**Figure 1:**
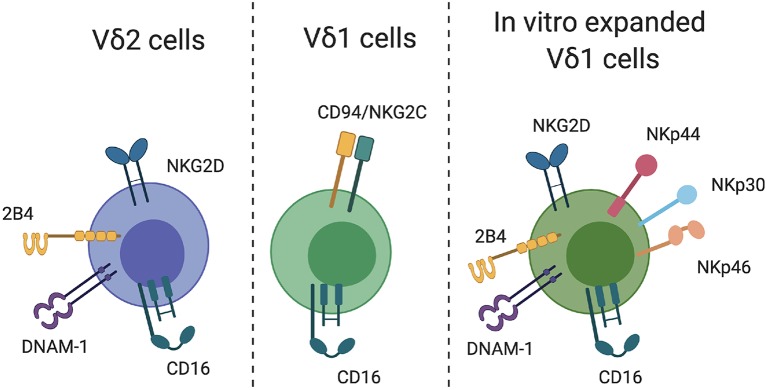
γδT cell recognition of target cells. Relevant receptors that may facilitate γδT cell recognition of HIV-infected CD4 T cells are indicated for Vδ2, Vδ1, and *in vitro*-expanded Vδ1 cells.

Perhaps the strongest proof-of-concept evidence for Vδ2-mediated elimination of infected CD4+ T cells following latency reversal *in vitro* was reported by Garrido et al. (2018b). Despite low frequencies of Vδ2 cells in ART-treated donors *ex vivo*, pamidronate + IL-2 treatment successfully expanded the Vδ2 population in a manner comparable to that of uninfected donors *in vitro* (i.e., a similar fold-increase). The expanded cells expressed CD56 and CD16, as well as relatively low levels of the inhibitory markers PD-1 and CTLA-4. Interestingly, both *ex vivo* isolated and *in vitro* expanded Vδ2 cells were equally capable of inhibiting HIV replication in autologous superinfected CD4+ T cells, with a level of inhibition comparable to that of CD8+ CTL. More importantly, however, expanded Vδ2 cells degranulated in response to co-culture with HIV-infected, but not uninfected, CD4+ T cells. These results were further extended to a latency clearance assay, which demonstrated that expanded Vδ2 cells cleared infected CD4+ T cells derived from ART-treated donors following *in vitro* reactivation with vorinostat. This study provides promising evidence for the ability of Vδ2 cells to contribute to an HIV cure approach that relies on eliminating reactivated virally infected target cells. A number of important questions remain, however, particularly regarding the mechanisms of Vδ2 recognition of HIV-infected cells as well as the optimal conditions for expansion Vδ2 cells with the most potent cytotoxicity. The need to address these gaps in knowledge is evidenced in a recent study by James et al. (2020) which found that the presence of gamma delta T cells in a viral outgrowth assay impacted viral replication in 4 of 15 donors. CD16 expression on Vδ2 cells correlated with a reduction in HIV recovery, but the key receptors involved in recognition of CD4 target cells and the reason for the heterogeneity in responses among the cohort remains unknown. Below, we explore the potential mechanisms that could be exploited in a gamma delta T-mediated HIV cure strategy (summarized in Figure 1).

## Receptors Mediating Direct Cytotoxicity

Vδ2 cells express a number of receptors that might facilitate recognition of HIV-infected T cells. A critical receptor for the detection and killing of transformed and virally infected is NKG2D, which is commonly expressed on NK cells. NKG2D is widely expressed by Vδ2 cells both *ex vivo* and following *in vitro* expansion, and can mediate direct killing of target cells (Rincon-Orozco et al., 2005; Wrobel et al., 2007). The NKG2D ligands ULBP-1,−2, and−3 are absent on uninfected CD4+ T cells, but are highly upregulated upon HIV infection and mediate NK-cell killing of HIV-infected cells (Ward et al., 2007). NKG2D is therefore a strong candidate for Vδ2-mediated direct recognition and killing of reactivated HIV-infected cells. NK cells can also recognize infected CD4+ T cells through 2B4-CD48 binding (Ward et al., 2007). Although 2B4 is highly expressed on Vδ2 cells *ex vivo*, triggering of 2B4 in isolation is insufficient to activate γδT effector functions (Nakajima et al., 1999). Whether phosphoantigen-expanded Vδ2 cells can be activated through 2B4 signaling and/or recognition of CD48 on HIV-infected T cells is currently unknown. Similarly, there is a potential for Vδ2-expressed DNAM-1 to recognize its ligand PVR on infected CD4+ T cells, although NK cell killing of infected cells through this mechanism seems to require signaling through both NKG2D and DNAM-1, rather than DNAM-1 alone (Cifaldi et al., 2019). In general, it will be important for future studies to consider the impact of activating receptor ligation both among *ex vivo*-derived Vδ2 cells, as well as *in vitro* expanded cells. For example, NKG2D ligation on resting Vδ2 cells is reported to have either no or minimal functional impact (Rincon-Orozco et al., 2005; Nedellec et al., 2010), but in the context of TCR stimulation can provide robust co-stimulatory activity and effector function (Nedellec et al., 2010).

Expression of cytotoxicity receptors on Vδ1 cells differs substantially from those described on Vδ2 cells. Vδ1 cells commonly express CD94 paired with either the inhibitory receptor NKG2A (CD159a) or the activating receptor NKG2C (CD159c). Chronic HIV infection is associated with a significant increase in NKG2C expression on Vδ1 cells, which can substantially boost Vδ1 cell cytotoxicity when triggered by binding to its ligand HLA-E (Fausther-Bovendo et al., 2008). Interestingly, Vδ1 cells derived *ex vivo* from HIV-infected participants can lyse autologous HIV-infected CD4+ T cells in a manner which is partially dependent on Vδ1 NKG2C expression (Fausther-Bovendo et al., 2008), suggesting a currently unrealized potential for Vδ1 cells in anti-HIV immunotherapy. The *in vitro* expansion of Vδ1 cells is less commonly reported or standardized compared to Vδ2 cells, largely due to the lack of an antigen that can easily stimulate the majority of Vδ1 cells *ex vivo*. Several groups, however, have reported protocols to expand these cells using a combination of γδ TCR stimulus and cytokines. Almeida et al. reported on the production of “Delta One T (DOT)” cells using isolated γδT cells, CD3 engagement and a cocktail of cytokines, which resulted in a 60,000-fold expansion of Vδ1 cells in culture (Almeida et al., 2016). Vδ1 expansion can also be induced through the culture of isolated γδT cells with PHA and IL-7 (Wu et al., 2015). In both studies, expanded Vδ1 cells expressed a number of surface markers related to cytotoxic function, including NKp30, NKp44, NKp46, NKG2D, 2B4, DNAM-1, and CD94. Other approaches to expand Vδ1 cells include the use of artificial APCs (aAPCs) presenting CMV-derived peptides (Polito et al., 2019). Vδ1 cells expanded by this approach recognize an array of tumor cells and virally infected cell lines, but the mechanism underlying this recognition was not assessed. Although the translational potential of DOT cells and other expanded Vδ1 cells are currently focussed on cancer immunotherapy, the expression of traditionally NK cell-associated markers such as NKp44, NKG2D, and 2B4 on expanded Vδ1 cells may also mediate recognition of HIV-infected CD4+ T cells (Ward et al., 2007).

## Antibody-Dependent Cytotoxicity

In addition to direct recognition and killing of HIV-infected target cells, HIV-specific antibodies can mediate antibody-dependent cellular cytotoxicity (ADCC) via Fc receptor-expressing cells such as NK cells. Naturally occurring anti-HIV ADCC antibodies decline following ART (Madhavi et al., 2015, 2017) and are not boosted following panobinostat administration (as a latency-reversing agent, LRA) and subsequent analytical treatment interruption (ATI) (Lee et al., 2017). This suggests such antibodies may be insufficient to mediate the level of ADCC needed to clear the latent reservoir. The infusion of more potent broadly neutralizing antibodies (BnAbs), however, may be sufficient to allow FcR-expressing cells to recognize Env expression on reactivated infected cells (Bruel et al., 2016). HIV-specific ADCC has been widely studied in the context of NK cells, but less so in relation to unconventional T cells. Both Vδ1 and Vδ2 cells can express FcgRIIIA (CD16) (Angelini et al., 2004; Couzi et al., 2012; He et al., 2013), making them potential mediators of ADCC in the context of latency reactivation and BnAb therapy.

Despite depletion of Vδ2 cells during chronic HIV infection, ART-treated individuals exhibit similar frequencies of CD16+ Vδ2 cells compared to uninfected controls (although there is intra-donor variability in steady-state CD16 expression) (He et al., 2013). As such, *ex vivo* CD16-mediated degranulation responses are comparable between these groups (He et al., 2013), suggesting that primary Vδ2 cells from ART-treated individuals are capable of performing ADCC. Poonia and Pauza (2012) demonstrated that zoledronate can expand Vδ2 cells *in vitro* from both HIV-uninfected and HIV-infected donors, with expression of CD16 detectible on a subset of the expanded cells. Although expanded Vδ2 cells from HIV-infected donors exhibited lower ADCC than those from uninfected donors, they remained capable of degranulation and cytokine expression following CD16 cross-linking.

The expression of CD16 on Vδ1 cells has been studied in the context of HCMV infection (Couzi et al., 2012; Bachelet et al., 2014) and cancer immunotherapy (Fisher et al., 2014), but despite the high prevalence of HCMV seropositivity among HIV-infected populations (Gianella and Letendre, 2016), there has been little study of Vδ1 ADCC in the context of HIV infection. The expanded population of Vδ1 cells in HIV-infected individuals exhibits high expression of perforin (Boullier et al., 1997), but whether this translates into antibody-mediated cytotoxicity is unknown. Similar gaps in knowledge exist for *in vitro*-expanded Vδ1 cells. There is no data available to describe CD16 expression on DOT cells (Almeida et al., 2016), while PHA/IL-7-expanded Vδ1 cells express moderate levels of CD16 and high levels of GzmB and Perforin, though they have not been assessed for ADCC activity (Wu et al., 2015).

## Approaches for Gamma Delta Immunotherapy

As described above, there are many *in vitro* protocols that result in Vd2 or Vd1 T cell expansion from bulk PBMC. To translate these ideas to the clinic, there are two major approaches that can be considered: *in vivo* gamma delta T cell expansion, or adoptive transfer of *in vitro*-expanded cells. Currently, *in vivo* expansion has only been demonstrated for human Vd2 T cells. This method involves the administration of zoledronate and recombinant IL-2, and has successfully expanded Vd2 T cells in HIV-infected participants in a small clinical trial (Poccia et al., 2009). An advantage of this approach is that it is relatively simple and involves an existing, clinically approved drug (under the brand name Zometa). It would not, however, likely be suitable for individuals with substantial Vd2 T cell depletion/exhaustion or Vd2 T cell phenotypes that lack the receptors determined to be key in recognizing HIV-infected target cells. In such instances, it may be more attractive to consider the use of “off-the-shelf,” *in vitro*-expanded gamma delta T cells derived from HIV uninfected donors. Such cells could be expanded by any of the protocols described above, and could include both Vd2 and Vd1 T cell subsets. This approach has been tested in clinical trials for cancer patients (Lo Presti et al., 2017) and also used in studies of non-human primate Vd2 T cells (Qaqish et al., 2017).

## Accessing the Latent HIV Reservoir

While *in vitro* studies can provide an important assessment of cytotoxic capacity and recognition of infected T cells, they largely ignore one of the major barriers to achieving an HIV cure: the anatomical localization of the latent reservoir. Recent evidence suggests that the B cell follicle is a key site of HIV replication, even during ART (Bronnimann et al., 2018). Germinal center (GC) T follicular helper (Tfh) cells are highly permissive to HIV infection (Kohler et al., 2016; Aid et al., 2018), while follicular dendritic cells (FDCs) can capture HIV virions and maintain an infectious virus reservoir even during ART (Smith et al., 2001; Bronnimann et al., 2018). A major barrier to the clearance of virus from the B cell follicle is the fact that the follicle is partially protected from CTL activity. Of the few CTLs found in the follicle, many may be exhausted or exhibit a regulatory phenotype (Bronnimann et al., 2018). Similarly, there are few NK cells found in the human lymph node, and a high proportion of LN NK cells in HIV-infected individuals exhibit an anergic CD56-CD16+ phenotype (Luteijn et al., 2011). Given these limitations, expanded γδT cells may be strong candidates for B cell follicle targeting and elimination of infected cells. Vδ2 cells can acquire a Tfh-like phenotype, complete with expression of the GC-homing marker CXCR5 (Caccamo et al., 2006; Bansal et al., 2012), and such cells have been described both *in vitro* and *in vivo* in human lungs (Caccamo et al., 2006). Furthermore, γδT cells have been visualized within GCs in the gastrointestinal mucosa, lymph nodes, tonsil and spleen, likely due to the induction of a LN migration program that occurs following Vδ2 TCR triggering (Brandes et al., 2003). Functional studies of Vδ2 Tfh cells have largely focused on their capacity to provide B cell help, leaving open the question of their cytotoxic potential and expression of cytotoxicity receptors. Nonetheless, the fact that γδT cells appear to access the B cell follicle merits further study of their potential to eliminate HIV-infected cells in this unique environment (Bronnimann et al., 2018).

Beyond the B cell follicle, many other tissues serve as HIV reservoir sites, including the spleen, bone marrow, liver, gut, nervous system, lung, and male and female reproductive tracts (Wong and Yukl, 2016; Cantero-Perez et al., 2019). Infection of tissue-resident T cells may be particularly important, given their longevity and capacity for self-renewal (Cantero-Perez et al., 2019). Both expanded Vδ1 and Vδ2 cells appear to be excellent candidates for tissue trafficking *in vivo*. When administered to mice, human-derived DOT cells seeded, and then further expanded in, various tissues including the spleen, lung, bone marrow, and liver (Almeida et al., 2016). The tissue-resident cells retained function, as assessed by IFNg and TNF production, and exhibited evidence of ongoing proliferation. Similarly, the adoptive transfer of expanded Vδ2 cells has been studied in non-human primates. Infusion of zoledronate-expanded Vδ2 cells resulted in trafficking to and persistence in the airway for at least 7 days (Qaqish et al., 2017).

## Potential Advantages and Disadvantages of γδT Cell Immunotherapy

As with any immune-based intervention, there are both key advantages offered by γδT cells over other T cell therapies and issues that will need to be addressed prior to clinical testing.

Advantages of γδT cell-based immunotherapy include:

1) *Lack of MHC restriction*: GMP-compliant protocols have been developed to generate large banks of expanded γδT cells, allowing for an “off-the-shelf” allogeneic product that is not reliant on MHC matching (Deniger et al., 2014; Almeida et al., 2016; Polito et al., 2019). The lack of MHC-restriction of γδT cells is a major advantage that should avoid issues of graft-vs.-host disease in γδT -based immunotherapy. Indeed, adoptive transfer of haploidentical expanded γδT cells from the family members of cancer patients proved to be safe and highly effective toward achieving complete remission (Wilhelm et al., 2014).

2) *Safety*: Clinical trials of human γδT cell therapy, either using *in vivo* Vδ2 expansion or adoptive transfer of expanded Vδ2 cells, have proven to be safe and well-tolerated (Lo Presti et al., 2017). A major disadvantage to current αβ T cell-based chimeric antigen receptor (CAR)-T cell therapies is the potential for serious and/or fatal side effects, including cytokine release syndrome (van Den Berg et al., 2015), off-target effects due to antigen cross-reactivity (Linette et al., 2013), or transgenic TCR mispairing with endogenous TCR (Zhang et al., 2004). Several studies suggest that γδT-based immunotherapy is less likely to result in cytokine release syndrome (CRS) or off-target effects than αβ T cells (Harrer et al., 2017; Pauza et al., 2018; Rotolo et al., 2019). Indeed, expanded γδ T cells can mediate potent cytotoxicity but simultaneously produce lower levels of cytokines than αβ T cells (Harrer et al., 2017).

3) *HIV-associated expansion and differentiation of Vd1 cells*: As discussed in the introduction, Vδ1 cell frequency and absolute counts increase in the circulation during HIV infection. This increase persists during suppressive ART, resulting in a pool of terminally differentiated Vδ1 cells (Olson et al., 2018) that express NCRs such as NKG2C (Fausther-Bovendo et al., 2008). To date, we are not aware of any studies that have assessed the expansion and subsequent anti-HIV cytotoxicity of Vδ1 cells specifically derived from HIV-infected individuals. If expansion of Vδ1 cells from healthy donors does not induce the expression of NKG2C or other NCRs that are upregulated during HIV infection, then it may be advantageous to isolate and expand Vδ1 cells expressing these markers from people living with HIV. Care must be taken, however, to characterize the pro-inflammatory nature of these cells, as discussed below.

Potential challenges to γδT cell-based immunotherapy include:

1) *In vivo potency*: One of the major challenges facing the implementation of γδT cell therapy in cancer is a discrepancy between *in vitro* and *in vivo* γδT cell potency (Pauza et al., 2018). *In vitro*, γδT cell cytotoxicity can be achieved with effector:target ratios of <1, but results of human clinical trials show considerably more heterogeneity in objective responses to γδT immunotherapy (Pauza et al., 2018). Pre-clinical animal studies are hindered by the fact that Vδ2Vg9 phosphoantigen-reactive T cells are found only in humans and non-human primates (NHP). Studies in mouse models are therefore less informative and have more caveats than studies of other lymphocyte subsets. In the context of HIV infection, it will be critical to move studies into NHPs and SIV infection prior to human clinical trials.

2) γδ*T cell infection by HIV*: Typically, both Vδ1 and Vδ2 cells lack expression of the HIV co-receptor CD4, seemingly rendering them refractory to direct infection. Concerningly, however, Soriano-Sarabia et al. (2015) reported that replication-competent HIV could be recovered from purified Vδ2 cells in 14 of 18 long-term ART recipients. Thus, the use of expanded autologous Vδ2 cells from HIV-infected patients may risk the reactivation and replication of a latent reservoir. This could be mitigated by the use of haploidentical or third-party Vδ2 cells from HIV-uninfected donors. Even in that scenario, though, consideration must be given to whether large numbers of adoptively transferred Vδ2 cells could be infected by HIV, since γδ immunotherapy would be reliant on the use of LRAs to reactivate the latent reservoir. Several reports now suggest that Vδ2 cells can transiently express CD4 during activation (Imlach et al., 2003; Soriano-Sarabia et al., 2015; Strbo et al., 2016) and can be productively infected with HIV (Wallace et al., 1997), which likely mediates the establishment of the latent reservoir detected by Soriano-Sarabia. For that reason, *in vitro* Vδ2 expansion and adoptive transfer may be preferable compared to *in vivo* Vδ2 expansion using aminobisphosphonate drugs.

3) *Depletion of V*δ*2 cells*: In contrast to Vδ1 cells, Vδ2 cells are depleted during HIV infection and do not fully recover following ART (as discussed previously). Most data suggest that Vδ2 cells expand *in vitro* to a similar extent regardless of the HIV status of the donor, but individuals who exhibit low Vδ2 frequencies *ex vivo* will produce fewer absolute numbers of Vδ2 cells after expansion (Garrido et al., 2018b). Furthermore, the observation that the ADCC activity of Vδ2 cells derived from ART-treated donors was reduced compared to uninfected donors (Poonia and Pauza, 2012) suggests that further attention needs to be paid to functional defects of Vδ2 cells in HIV-infected donors.

4) *Contribution to immune activation*: While the causes of Vδ1 expansion during HIV infection are not conclusively known, it is likely that microbial translocation and loss of epithelial barrier integrity in the gut mucosa drive activation and differentiation of this subset (Harris et al., 2010; Olson et al., 2018). Some studies have shown that Vδ1 cells exhibit proinflammatory cytokine production that correlates with CD8+ T cell activation (Olson et al., 2018) or killing of bystander CD4+ T cells (Sindhu et al., 2003) during HIV infection. Thorough assessment of the characteristics of expanded Vδ1 cells derived from HIV-infected donors will be required to determine the potential pathogenic impact of these cells if adoptively transferred.

## Concluding Thoughts and Future Directions

Intense interest in γδT cell-based immunotherapy for cancer is continually driving innovative and novel approaches to harness γδT cell cytotoxicity, which should be considered in the context of HIV cure strategies. For example, despite substantial differences in antigen specificity, NK cell receptor expression, and tissue tropism between Vδ1 and Vδ2 cells, it may be more effective to non-specifically expand a diverse population of γδT cells than to focus on the development of pure Vδ1 or Vδ2 populations. Using aAPCs engineered to express a number of co-stimulatory molecules, Deniger et al. (2014) demonstrated the expansion of a mixed population of Vδ1, Vδ2, and Vδ1-Vδ2- cells that recognized tumor cells via NKG2D, DNAM-1, and the γδ TCR. In an *in vivo* murine model, this mixed population was more effective at eliminating ovarian cancer xenografts than any of the individual γδT subsets alone. Similar results were obtained with mixed γδT cells in a model of neuroblastoma, where Vδ2 cells mediated CD16-dependent ADCC and Vd1 cells exhibited direct cytotoxicity (Fisher et al., 2014). Given the utility of both ADCC-based and direct recognition of HIV-infected T cells in cure strategies, mixed γδT expansion may be a potent method by which to generate effector γδT cells. More complex approaches could also engineer such cells to express a CAR, providing an additional mechanism by which to recognize infected cells (Rotolo et al., 2019).

Regardless of the nature of the γδT cells chosen for study, the path forward for γδT immunotherapy in HIV cure is clear: First, incisive mechanistic studies are needed to establish the most efficient mechanisms of γδT recognition of reactivated HIV-infected target cells. Despite the intriguing results of Garrido et al. (2018b), the latency-clearing capacity of Vδ2 cells needs to be replicated and the mechanism of recognition defined. With this information, it may be possible to identify γδT cell subsets whose selective expansion would generate better effector cell populations compared to bulk expansion protocols. Second, *in vivo* animal studies using non-human primates will be critical to addressing questions of tissue targeting and assessment of *in vivo* potency. Reagents exist to characterize both Vδ1 and Vδ2 cells in NHPs, and studies confirm that these cells recapitulate many aspects of human immunology including expression of CD16 (Hodara et al., 2014), NKG2D, NKG2A, GzmB, and CD107a (Tuero et al., 2016), as well as the ability to kill SIV-infected cells (Malkovsky et al., 1992; Wallace et al., 1994). SIV-infected macaques also recapitulate important aspects of HIV-infected human cohorts, including inversion of the Vδ1:Vδ2 ratio and microbial translocation-induced expansion of Vδ1 cells (Harris et al., 2010). Critically, translational studies of immunity to *Mycobacterium tuberculosis* have demonstrated the feasibility of both expanding macaque Vδ2 cells *in vivo* (Ali et al., 2007) or adoptively transferring *in vitro* expanded cells (Qaqish et al., 2017). With these goals in mind, the application of γδT-based immunotherapy to HIV cure strategies represents an exciting and informative research avenue.

## Author Contributions

JJ and SK wrote and edited the manuscript.

## Conflict of Interest

The authors declare that the research was conducted in the absence of any commercial or financial relationships that could be construed as a potential conflict of interest.

## References

[B1] Aid M. Dupuy F. P. Moysi E. Moir S. Haddad E. K. Estes J. D.. (2018). Follicular CD4 T helper cells as a major HIV reservoir compartment: a molecular perspective. Front. Immunol. 9:895. 10.3389/fimmu.2018.00895 29967602 PMC6015877

[B2] Ali Z. Shao L. Halliday L. Reichenberg A. Hintz M. Jomaa H.. (2007). Prolonged (E)-4-hydroxy-3-methyl-but-2-enyl pyrophosphate-driven antimicrobial and cytotoxic responses of pulmonary and systemic Vγ2Vδ2 T cells in macaques. J. Immunol. 179, 8287–8296. 10.4049/jimmunol.179.12.8287 18056373 PMC2865221

[B3] Almeida A. R. Correia D. V. Fernandes-Platzgummer A. Da Silva C. L. Da Silva M. G. Anjos D. R.. (2016). Δ one T cells for immunotherapy of chronic lymphocytic leukemia: clinical-grade expansion/differentiation and preclinical proof of concept. Clin. Cancer Res. 22, 5795–5804. 10.1158/1078-0432.CCR-16-0597 27307596

[B4] Angelini D. F. Borsellino G. Poupot M. Diamantini A. Poupot R. Bernardi G.. (2004). FcγRIII discriminates between 2 subsets of Vγ9Vδ2 effector cells with different responses and activation pathways. Blood 104, 1801–1807. 10.1182/blood-2004-01-0331 15178578

[B5] Bachelet T. Couzi L. Pitard V. Sicard X. Rigothier C. Lepreux S.. (2014). Cytomegalovirus-responsive γδ T cells: novel effector cells in antibody-mediated kidney allograft microcirculation lesions. J. Am. Soc. Nephrol. 25, 2471–2482. 10.1681/ASN.2013101052 24744438 PMC4214528

[B6] Bansal R. R. Mackay C. R. Moser B. Eberl M. (2012). IL-21 enhances the potential of human γδ T cells to provide B-cell help. Eur. J. Immunol. 42, 110–119. 10.1002/eji.201142017 22009762

[B7] Bhatnagar N. Girard P. M. Lopez-Gonzalez M. Didier C. Collias L. Jung C.. (2017). Potential role of Vδ2(+) γδ T cells in regulation of immune activation in primary HIV infection. Front. Immunol. 8:1189. 10.3389/fimmu.2017.01189 28993778 PMC5622291

[B8] Boullier S. Dadaglio G. Lafeuillade A. Debord T. Gougeon M. L. (1997). V δ 1 T cells expanded in the blood throughout HIV infection display a cytotoxic activity and are primed for TNF-alpha and IFN-γ production but are not selected in lymph nodes. J. Immunol. 159, 3629–3637. 9317163

[B9] Brandes M. Willimann K. Lang A. B. Nam K. H. Jin C. Brenner M. B.. (2003). Flexible migration program regulates γ δ T-cell involvement in humoral immunity. Blood 102, 3693–3701. 10.1182/blood-2003-04-1016 12881309

[B10] Bronnimann M. P. Skinner P. J. Connick E. (2018). The B-cell follicle in HIV infection: barrier to a cure. Front. Immunol. 9:20. 10.3389/fimmu.2018.00020 29422894 PMC5788973

[B11] Bruel T. Guivel-Benhassine F. Amraoui S. Malbec M. Richard L. Bourdic K.. (2016). Elimination of HIV-1-infected cells by broadly neutralizing antibodies. Nat. Commun. 7:10844. 10.1038/ncomms10844 26936020 PMC4782064

[B12] Caccamo N. Battistini L. Bonneville M. Poccia F. Fournie J. J. Meraviglia S.. (2006). CXCR5 identifies a subset of Vγ9Vδ2 T cells which secrete IL-4 and IL-10 and help B cells for antibody production. J. Immunol. 177, 5290–5295. 10.4049/jimmunol.177.8.5290 17015714

[B13] Cantero-Perez J. Grau-Exposito J. Serra-Peinado C. Rosero D. A. Luque-Ballesteros L. Astorga-Gamaza A.. (2019). Resident memory T cells are a cellular reservoir for HIV in the cervical mucosa. Nat. Commun. 10:4739. 10.1038/s41467-019-12732-2 31628331 PMC6802119

[B14] Chevalier M. F. Bhatnagar N. Didier C. Lopez-Gonzalez M. Pavie J. Bollens D. (2019). γδ T-cell subsets in HIV controllers: potential role of T γδ 17 cells in the regulation of chronic immune activation. Aids 33, 1283–1292. 10.1097/QAD.0000000000002196 30870199

[B15] Cifaldi L. Doria M. Cotugno N. Zicari S. Cancrini C. Palma P.. (2019). DNAM-1 Activating receptor and its ligands: how do viruses affect the NK cell-mediated immune surveillance during the various phases of infection? Int. J. Mol. Sci. 20:3715. 10.3390/ijms20153715 31366013 PMC6695959

[B16] Cimini E. Agrati C. D'offizi G. Vlassi C. Casetti R. Sacchi A.. (2015). Primary and chronic HIV infection differently modulates mucosal Vδ1 and Vδ2 T-cells differentiation profile and effector functions. PLoS ONE 10:e0129771. 10.1371/journal.pone.0129771 26086523 PMC4472518

[B17] Couzi L. Pitard V. Sicard X. Garrigue I. Hawchar O. Merville P.. (2012). Antibody-dependent anti-cytomegalovirus activity of human γδ T cells expressing CD16 (FcγRIIIa). Blood 119, 1418–1427. 10.1182/blood-2011-06-363655 22180442

[B18] Davey M. S. Willcox C. R. Hunter S. Oo Y. H. Willcox B. E. (2018). Vδ2(+) T cells-two subsets for the price of one. Front. Immunol. 9:2106. 10.3389/fimmu.2018.02106 30319605 PMC6167451

[B19] Davey M. S. Willcox C. R. Joyce S. P. Ladell K. Kasatskaya S. A. Mclaren J. E.. (2017). Clonal selection in the human Vδ1 T cell repertoire indicates γδ TCR-dependent adaptive immune surveillance. Nat. Commun. 8:14760. 10.1038/ncomms14760 28248310 PMC5337994

[B20] De Maria A. Ferrazin A. Ferrini S. Ciccone E. Terragna A. Moretta L. (1992). Selective increase of a subset of T cell receptor γ δ T lymphocytes in the peripheral blood of patients with human immunodeficiency virus type 1 infection. J. Infect. Dis. 165, 917–919. 10.1093/infdis/165.5.917 1533237

[B21] De Paoli P. Gennari D. Martelli P. Basaglia G. Crovatto M. Battistin S.. (1991). A subset of γ δ lymphocytes is increased during HIV-1 infection. Clin. Exp. Immunol. 83, 187–191. 10.1111/j.1365-2249.1991.tb05612.x 1825186 PMC1535251

[B22] Deng K. Pertea M. Rongvaux A. Wang L. Durand C. M. Ghiaur G.. (2015). Broad CTL response is required to clear latent HIV-1 due to dominance of escape mutations. Nature 517, 381–385. 10.1038/nature14053 25561180 PMC4406054

[B23] Deniger D. C. Maiti S. N. Mi T. Switzer K. C. Ramachandran V. Hurton L. V.. (2014). Activating and propagating polyclonal γ δ T cells with broad specificity for malignancies. Clin. Cancer Res. 20, 5708–5719. 10.1158/1078-0432.CCR-13-3451 24833662 PMC4233015

[B24] Enders P. J. Yin C. Martini F. Evans P. S. Propp N. Poccia F.. (2003). HIV-mediated γδ T cell depletion is specific for Vγ2+ cells expressing the Jγ1.2 segment. AIDS Res. Hum. Retrovir. 19, 21–29. 10.1089/08892220360473934 12581513

[B25] Fausther-Bovendo H. Wauquier N. Cherfils-Vicini J. Cremer I. Debre P. Vieillard V. (2008). NKG2C is a major triggering receptor involved in the V[δ]1 T cell-mediated cytotoxicity against HIV-infected CD4 T cells. Aids 22, 217–226. 10.1097/QAD.0b013e3282f46e7c 18097224

[B26] Fehniger T. A. Herbein G. Yu H. Para M. I. Bernstein Z. P. O'brien W. A.. (1998). Natural killer cells from HIV-1+ patients produce C-C chemokines and inhibit HIV-1 infection. J. Immunol. 161, 6433–6438. 9834136

[B27] Fichtner A. S. Karunakaran M. M. Gu S. Boughter C. T. Borowska M. T. Starick L.. (2020). Alpaca (*Vicugna pacos*), the first nonprimate species with a phosphoantigen-reactive Vγ9Vδ2 T cell subset. Proc. Natl. Acad. Sci. U.S.A. 117, 6697–6707. 10.1073/pnas.1909474117 32139608 PMC7104304

[B28] Fisher J. P. Yan M. Heuijerjans J. Carter L. Abolhassani A. Frosch J.. (2014). Neuroblastoma killing properties of Vδ2 and Vδ2-negative γδT cells following expansion by artificial antigen-presenting cells. Clin. Cancer Res. 20, 5720–5732. 10.1158/1078-0432.CCR-13-3464 24893631 PMC4445920

[B29] Garrido C. Abad-Fernandez M. Tuyishime M. Pollara J. J. Ferrari G. Soriano-Sarabia N.. (2018a). Interleukin-15-stimulated natural killer cells clear HIV-1-infected cells following latency reversal *ex vivo*. J. Virol. 92:JVI.00235-18. 10.1128/JVI.00235-18 29593039 PMC5974478

[B30] Garrido C. Clohosey M. L. Whitworth C. P. Hudgens M. Margolis D. M. Soriano-Sarabia N. (2018b). Γδ T cells: an immunotherapeutic approach for HIV cure strategies. JCI Insight 3:e120121. 10.1172/jci.insight.120121 29925697 PMC6124426

[B31] Gianella S. Letendre S. (2016). Cytomegalovirus and HIV: a Dangerous Pas de Deux. J. Infect. Dis. 214(Suppl. 2), S67–74. 10.1093/infdis/jiw217 27625433 PMC5021239

[B32] Godfrey D. I. Le Nours J. Andrews D. M. Uldrich A. P. Rossjohn J. (2018). Unconventional T cell targets for cancer immunotherapy. Immunity 48, 453–473. 10.1016/j.immuni.2018.03.009 29562195

[B33] Groh V. Steinle A. Bauer S. Spies T. (1998). Recognition of stress-induced MHC molecules by intestinal epithelial γδ T cells. Science 279, 1737–1740. 10.1126/science.279.5357.1737 9497295

[B34] Gupta R. K. Peppa D. Hill A. L. Galvez C. Salgado M. Pace M.. (2020). Evidence for HIV-1 cure after CCR5Δ32/Δ32 allogeneic haemopoietic stem-cell transplantation 30 months post analytical treatment interruption: a case report. Lancet HIV. 7, e340–347. 10.1016/S2352-3018(20)30069-2 32169158 PMC7606918

[B35] Gutierrez-Arcelus M. Teslovich N. Mola A. R. Polidoro R. B. Nathan A. Kim H.. (2019). Lymphocyte innateness defined by transcriptional states reflects a balance between proliferation and effector functions. Nat. Commun. 10:687. 10.1038/s41467-019-08604-4 30737409 PMC6368609

[B36] Harrer D. C. Simon B. Fujii S. I. Shimizu K. Uslu U. Schuler G.. (2017). RNA-transfection of γ/δ T cells with a chimeric antigen receptor or an alpha/beta T-cell receptor: a safer alternative to genetically engineered alpha/beta T cells for the immunotherapy of melanoma. BMC Cancer 17:551. 10.1186/s12885-017-3539-3 28818060 PMC5561563

[B37] Harris L. D. Klatt N. R. Vinton C. Briant J. A. Tabb B. Ladell K.. (2010). Mechanisms underlying γδ T-cell subset perturbations in SIV-infected Asian rhesus macaques. Blood 116, 4148–4157. 10.1182/blood-2010-05-283549 20660793 PMC2993620

[B38] Hayday A. C. (2000). [γ][δ] cells: a right time and a right place for a conserved third way of protection. Annu. Rev. Immunol. 18, 975–1026. 10.1146/annurev.immunol.18.1.975 10837080

[B39] He X. Liang H. Hong K. Li H. Peng H. Zhao Y.. (2013). The potential role of CD16+ Vγ2Vδ2 T cell-mediated antibody-dependent cell-mediated cytotoxicity in control of HIV type 1 disease. AIDS Res. Hum. Retrovir. 29, 1562–1570. 10.1089/aid.2013.0111 23957587 PMC3848486

[B40] Hinz T. Wesch D. Friese K. Reckziegel A. Arden B. Kabelitz D. (1994). T cell receptor γ δ repertoire in HIV-1-infected individuals. Eur. J. Immunol. 24, 3044–3049. 10.1002/eji.1830241219 7805732

[B41] Hodara V. L. Parodi L. M. Chavez D. Smith L. M. Lanford R. Giavedoni L. D. (2014). Characterization of γδT cells in naive and HIV-infected chimpanzees and their responses to T-cell activators *in vitro*. J. Med. Primatol. 43, 258–271. 10.1111/jmp.12115 24660852 PMC4232220

[B42] Hutter G. Nowak D. Mossner M. Ganepola S. Mussig A. Allers K.. (2009). Long-term control of HIV by CCR5 Δ32/Δ32 stem-cell transplantation. N Engl. J. Med. 360, 692–698. 10.1056/NEJMoa0802905 19213682

[B43] Hviid L. Akanmori B. D. Loizon S. Kurtzhals J. A. Ricke C. H. Lim A.. (2000). High frequency of circulating γ δ T cells with dominance of the v(δ)1 subset in a healthy population. Int. Immunol. 12, 797–805. 10.1093/intimm/12.6.797 10837407

[B44] Hviid L. Smith-Togobo C. Willcox B. E. (2019). Human Vδ1(+) T cells in the immune response to plasmodium falciparum infection. Front. Immunol. 10:259. 10.3389/fimmu.2019.00259 30837999 PMC6382743

[B45] Imlach S. Leen C. Bell J. E. Simmonds P. (2003). Phenotypic analysis of peripheral blood γδ T lymphocytes and their targeting by human immunodeficiency virus type 1 *in vivo*. Virology 305, 415–427. 10.1006/viro.2002.1759 12573587

[B46] James K. S. Trumble I. Clohosey M. L. Moeser M. Roan N. R. Adimora A. A.. (2020). Measuring the contribution of γδ T cells to the persistent HIV reservoir. Aids 34, 363–371. 10.1097/QAD.0000000000002434 31764074 PMC6994336

[B47] Juno J. A. Eriksson E. M. (2019). γδ T-cell responses during HIV infection and antiretroviral therapy. Clin. Transl. Immunol. 8:e01069. 10.1002/cti2.1069 31321033 PMC6636517

[B48] Kohler S. L. Pham M. N. Folkvord J. M. Arends T. Miller S. M. Miles B.. (2016). Germinal center T follicular helper cells are highly permissive to HIV-1 and alter their phenotype during virus replication. J. Immunol. 196, 2711–2722. 10.4049/jimmunol.1502174 26873986 PMC4779697

[B49] Le Nours J. Gherardin N. A. Ramarathinam S. H. Awad W. Wiede F. Gully B. S.. (2019). A class of γδ T cell receptors recognize the underside of the antigen-presenting molecule MR1. Science 366, 1522–1527. 10.1126/science.aav3900 31857486

[B50] Lee W. S. Kristensen A. B. Rasmussen T. A. Tolstrup M. Ostergaard L. Sogaard O. S.. (2017). Anti-HIV-1 ADCC antibodies following latency reversal and treatment interruption. J. Virol. 91:e00603-17. 10.1128/JVI.00603-17 28539449 PMC5512246

[B51] Li Z. Jiao Y. Hu Y. Cui L. Chen D. Wu H.. (2015). Distortion of memory Vδ2 γδ T cells contributes to immune dysfunction in chronic HIV infection. Cell Mol. Immunol. 12, 604–614. 10.1038/cmi.2014.77 25220734 PMC4579648

[B52] Li Z. Li W. Li N. Jiao Y. Chen D. Cui L.. (2014). γδ T cells are involved in acute HIV infection and associated with AIDS progression. PLoS ONE 9:e106064. 10.1371/journal.pone.0106064 25188438 PMC4154895

[B53] Linette G. P. Stadtmauer E. A. Maus M. V. Rapoport A. P. Levine B. L. Emery L.. (2013). Cardiovascular toxicity and titin cross-reactivity of affinity-enhanced T cells in myeloma and melanoma. Blood 122, 863–871. 10.1182/blood-2013-03-490565 23770775 PMC3743463

[B54] Lo Presti E. Pizzolato G. Gulotta E. Cocorullo G. Gulotta G. Dieli F.. (2017). Current advances in γδ T cell-based tumor immunotherapy. Front. Immunol. 8:1401. 10.3389/fimmu.2017.01401 29163482 PMC5663908

[B55] Lundqvist C. Baranov V. Hammarstrom S. Athlin L. Hammarstrom M. L. (1995). Intra-epithelial lymphocytes. Evidence for regional specialization and extrathymic T cell maturation in the human gut epithelium. Int. Immunol. 7, 1473–1487. 10.1093/intimm/7.9.1473 7495755

[B56] Luteijn R. Sciaranghella G. Van Lunzen J. Nolting A. Dugast A. S. Ghebremichael M. S.. (2011). Early viral replication in lymph nodes provides HIV with a means by which to escape NK-cell-mediated control. Eur. J. Immunol. 41, 2729–2740. 10.1002/eji.201040886 21630248 PMC8943700

[B57] Madhavi V. Ana-Sosa-Batiz F. E. Jegaskanda S. Center R. J. Winnall W. R. Parsons M. S.. (2015). Antibody-dependent effector functions against HIV decline in subjects receiving antiretroviral therapy. J. Infect. Dis. 211, 529–538. 10.1093/infdis/jiu486 25170105

[B58] Madhavi V. Kulkarni A. Shete A. Lee W. S. Mclean M. R. Kristensen A. B.. (2017). Effect of combination antiretroviral therapy on HIV-1-specific antibody-dependent cellular cytotoxicity responses in subtype B- and subtype C-infected cohorts. J. Acquir. Immune Defic. Syndr. 75, 345–353. 10.1097/QAI.0000000000001380 28346319

[B59] Malkovsky M. Bartz S. R. Mackenzie D. Radtke B. E. Wallace M. Manning J.. (1992). Are γ δ T cells important for the elimination of virus-infected cells? J. Med. Primatol. 21, 113–118. 1433261

[B60] Nakajima H. Cella M. Langen H. Friedlein A. Colonna M. (1999). Activating interactions in human NK cell recognition: the role of 2B4-CD48. Eur. J. Immunol. 29, 1676–1683. 10.1002/(SICI)1521-4141(199905)29:05<1676::AID-IMMU1676>3.0.CO;2-Y 10359122

[B61] Nedellec S. Sabourin C. Bonneville M. Scotet E. (2010). NKG2D costimulates human V gamma 9V delta 2 T cell antitumor cytotoxicity through protein kinase C theta-dependent modulation of early TCR-induced calcium and transduction signals. J. Immunol. 185, 55–63. 10.4049/jimmunol.1000373 20511557

[B62] Oliva A. Kinter A. L. Vaccarezza M. Rubbert A. Catanzaro A. Moir S.. (1998). Natural killer cells from human immunodeficiency virus (HIV)-infected individuals are an important source of CC-chemokines and suppress HIV-1 entry and replication *in vitro*. J. Clin. Invest. 102, 223–231. 10.1172/JCI2323 9649576 PMC509084

[B63] Olson G. S. Moore S. W. Richter J. M. Garber J. J. Bowman B. A. Rawlings C. A.. (2018). Increased frequency of systemic pro-inflammatory Vδ1(+) γδ T cells in HIV elite controllers correlates with gut viral load. Sci. Rep. 8:16471. 10.1038/s41598-018-34576-4 30405182 PMC6220338

[B64] Omi K. Shimizu M. Watanabe E. Matsumura J. Takaku C. Shinya E.. (2014). Inhibition of R5-tropic HIV type-1 replication in CD4(+) natural killer T cells by γδ T lymphocytes. Immunology 141, 596–608. 10.1111/imm.12221 24266436 PMC3956433

[B65] Pauza C. D. Cairo C. (2015). Evolution and function of the TCR Vγ9 chain repertoire: it's good to be public. Cell Immunol. 296, 22–30. 10.1016/j.cellimm.2015.02.010 25769734 PMC4466227

[B66] Pauza C. D. Liou M. L. Lahusen T. Xiao L. Lapidus R. G. Cairo C.. (2018). Γ δ T cell therapy for cancer: it is good to be local. Front Immunol 9, 1305. 10.3389/fimmu.2018.01305 29937769 PMC6003257

[B67] Pitard V. Roumanes D. Lafarge X. Couzi L. Garrigue I. Lafon M. E.. (2008). Long-term expansion of effector/memory Vδ2-γδ T cells is a specific blood signature of CMV infection. Blood 112, 1317–1324. 10.1182/blood-2008-01-136713 18539896 PMC2515135

[B68] Pizzolato G. Kaminski H. Tosolini M. Franchini D. M. Pont F. Martins F.. (2019). Single-cell RNA sequencing unveils the shared and the distinct cytotoxic hallmarks of human TCRVδ1 and TCRVδ2 γδ T lymphocytes. Proc. Natl. Acad. Sci. U.S.A. 116, 11906–11915. 10.1073/pnas.1818488116 31118283 PMC6576116

[B69] Poccia F. Battistini L. Cipriani B. Mancino G. Martini F. Gougeon M. L.. (1999). Phosphoantigen-reactive Vγ9Vδ2 T lymphocytes suppress *in vitro* human immunodeficiency virus type 1 replication by cell-released antiviral factors including CC chemokines. J. Infect. Dis. 180, 858–861. 10.1086/314925 10438380

[B70] Poccia F. Boullier S. Lecoeur H. Cochet M. Poquet Y. Colizzi V.. (1996). Peripheral V γ 9/V δ 2 T cell deletion and anergy to nonpeptidic mycobacterial antigens in asymptomatic HIV-1-infected persons. J. Immunol. 157, 449–461. 8683151

[B71] Poccia F. Cipriani B. Vendetti S. Colizzi V. Poquet Y. Battistini L.. (1997). CD94/NKG2 inhibitory receptor complex modulates both anti-viral and anti-tumoral responses of polyclonal phosphoantigen-reactive V gamma 9V delta 2 T lymphocytes. J. Immunol. 159, 6009–6017. 9550399

[B72] Poccia F. Gioia C. Martini F. Sacchi A. Piacentini P. Tempestilli M.. (2009). Zoledronic acid and interleukin-2 treatment improves immunocompetence in HIV-infected persons by activating Vgamma9Vδ2 T cells. Aids 23, 555–565. 10.1097/QAD.0b013e3283244619 19238075

[B73] Poggi A. Carosio R. Fenoglio D. Brenci S. Murdaca G. Setti M.. (2004). Migration of V δ 1 and V δ 2 T cells in response to CXCR3 and CXCR4 ligands in healthy donors and HIV-1-infected patients: competition by HIV-1 Tat. Blood 103, 2205–2213. 10.1182/blood-2003-08-2928elta 14630801

[B74] Poles M. A. Barsoum S. Yu W. Yu J. Sun P. Daly J.. (2003). Human immunodeficiency virus type 1 induces persistent changes in mucosal and blood γδ T cells despite suppressive therapy. J. Virol. 77, 10456–10467. 10.1128/JVI.77.19.10456-10467.2003 12970431 PMC228518

[B75] Polito V. A. Cristantielli R. Weber G. Del Bufalo F. Belardinilli T. Arnone C. M.. (2019). Universal ready-to-use immunotherapeutic approach for the treatment of Cancer: expanded and activated polyclonal γδ memory T cells. Front. Immunol. 10:2717. 10.3389/fimmu.2019.02717 31824502 PMC6883509

[B76] Poonia B. Pauza C. D. (2012). Γ δ T cells from HIV+ donors can be expanded *in vitro* by zoledronate/interleukin-2 to become cytotoxic effectors for antibody-dependent cellular cytotoxicity. Cytotherapy 14, 173–181. 10.3109/14653249.2011.623693 22029653

[B77] Provine N. M. Binder B. Fitzpatrick M. E. B. Schuch A. Garner L. C. Williamson K. D.. (2018). Unique and common features of innate-like human Vδ2(+) γδT cells and mucosal-associated invariant T cells. Front. Immunol. 9:756. 10.3389/fimmu.2018.00756 29740432 PMC5924964

[B78] Qaqish A. Huang D. Chen C. Y. Zhang Z. Wang R. Li S.. (2017). Adoptive transfer of phosphoantigen-specific γδ T cell subset attenuates mycobacterium tuberculosis infection in nonhuman primates. J Immunol. 198, 4753–4763. 10.4049/jimmunol.1602019 28526681 PMC5557270

[B79] Riedel D. J. Sajadi M. M. Armstrong C. L. Cummings J. S. Cairo C. Redfield R. R.. (2009). Natural viral suppressors of HIV-1 have a unique capacity to maintain γδ T cells. Aids 23, 1955–1964. 10.1097/QAD.0b013e32832ff1ff 19609200 PMC2956264

[B80] Rincon-Orozco B. Kunzmann V. Wrobel P. Kabelitz D. Steinle A. Herrmann T. (2005). Activation of V gamma 9V delta 2 T cells by NKG2D. J. Immunol. 175, 2144–2151. 10.4049/jimmunol.175.4.2144 16081780

[B81] Rossol R. Dobmeyer J. M. Dobmeyer T. S. Klein S. A. Rossol S. Wesch D. (1998). Increase in Vδ1+ γδ T cells in the peripheral blood and bone marrow as a selective feature of HIV-1 but not other virus infections. Br. J. Haematol. 100, 728–734. 10.1046/j.1365-2141.1998.00630.x 9531341

[B82] Rotolo R. Leuci V. Donini C. Cykowska A. Γitoni L. Medico G.. (2019). CAR-based strategies beyond T Lymphocytes: integrative opportunities for cancer adoptive immunotherapy. Int. J. Mol. Sci. 20:2839. 10.3390/ijms20112839 31212634 PMC6600566

[B83] Roy S. Ly D. Castro C. D. Li N. S. Hawk A. J. Altman J. D.. (2016). Molecular analysis of lipid-reactive Vδ1 γδ T cells identified by CD1c tetramers. J. Immunol. 196, 1933–1942. 10.4049/jimmunol.1502202 26755823 PMC4744554

[B84] Shan L. Deng K. Shroff N. S. Durand C. M. Rabi S. A. Yang H. C.. (2012). Stimulation of HIV-1-specific cytolytic T lymphocytes facilitates elimination of latent viral reservoir after virus reactivation. Immunity 36, 491–501. 10.1016/j.immuni.2012.01.014 22406268 PMC3501645

[B85] Silva-Santos B. Mensurado S. Coffelt S. B. (2019). γδ T cells: pleiotropic immune effectors with therapeutic potential in cancer. Nat. Rev. Cancer 19, 392–404. 10.1038/s41568-019-0153-5 31209264 PMC7614706

[B86] Simoes A. E. di Lorenzo B. Silva-Santos B. (2018). Molecular determinants of target cell recognition by human γδ T cells. Front. Immunol. 9:929. 10.3389/fimmu.2018.00929 29755480 PMC5934422

[B87] Sindhu S. T. Ahmad R. Morisset R. Ahmad A. Menezes J. (2003). Peripheral blood cytotoxic γδ T lymphocytes from patients with human immunodeficiency virus type 1 infection and AIDS lyse uninfected CD4+ T cells, and their cytocidal potential correlates with viral load. J. Virol. 77, 1848–1855. 10.1128/JVI.77.3.1848-1855.2003 12525619 PMC140951

[B88] Smith B. A. Gartner S. Liu Y. Perelson A. S. Stilianakis N. I. Keele B. F.. (2001). Persistence of infectious HIV on follicular dendritic cells. J. Immunol. 166, 690–696. 10.4049/jimmunol.166.1.690 11123354

[B89] Soriano-Sarabia N. Archin N. M. Bateson R. Dahl N. P. Crooks A. M. Kuruc J. D.. (2015). Peripheral Vγ9Vδ2 T cells are a novel reservoir of latent HIV infection. PLoS Pathog. 11:e1005201. 10.1371/journal.ppat.1005201 26473478 PMC4608739

[B90] Strbo N. Alcaide M. L. Romero L. Bolivar H. Jones D. Podack E. R.. (2016). Loss of intra-epithelial endocervical gamma delta (GD) 1 T cells in HIV-infected women. Am. J. Reprod. Immunol. 75, 134–145. 10.1111/aji.12458 26666220 PMC4715976

[B91] Tanaka Y. Morita C. T. Tanaka Y. Nieves E. Brenner M. B. Bloom B. R. (1995). Natural and synthetic non-peptide antigens recognized by human γ δ T cells. Nature 375, 155–158. 10.1038/375155a0 7753173

[B92] Triebel F. Faure F. Graziani M. Jitsukawa S. Lefranc M. P. Hercend T. (1988). A unique V-J-C-rearranged gene encodes a γ protein expressed on the majority of CD3+ T cell receptor-alpha/beta- circulating lymphocytes. J. Exp. Med. 167, 694–699. 10.1084/jem.167.2.694 2450164 PMC2188842

[B93] Tuero I. Venzon D. Robert-Guroff M. (2016). Mucosal and systemic γδ+ T cells associated with control of simian immunodeficiency virus infection. J. Immunol. 197, 4686–4695. 10.4049/jimmunol.1600579 27815422 PMC5136305

[B94] Tyler C. J. Doherty D. G. Moser B. Eberl M. (2015). Human Vγ9/Vδ2 T cells: Innate adaptors of the immune system. Cell Immunol. 296, 10–21. 10.1016/j.cellimm.2015.01.008 25659480

[B95] Uldrich A. P. Le Nours J. Pellicci D. G. Gherardin N. A. Mcpherson K. G. Lim R. T.. (2013). CD1d-lipid antigen recognition by the γδ TCR. Nat. Immunol. 14, 1137–1145. 10.1038/ni.2713 24076636

[B96] van Den Berg J. H. Gomez-Eerland R. van De Wiel B. Hulshoff L. Van Den Broek D. Bins A.. (2015). Case report of a fatal serious adverse event upon administration of T cells transduced With a MART-1-specific T-cell receptor. Mol. Ther. 23, 1541–1550. 10.1038/mt.2015.60 25896248 PMC4817886

[B97] van Der Heiden M. Bjorkander S. Rahman Qazi K. Bittmann J. Hell L. Jenmalm M. C.. (2020). Characterization of the γδ T-cell compartment during infancy reveals clear differences between the early neonatal period and 2 years of age. Immunol. Cell Biol. 98, 79–87. 10.1111/imcb.12303 31680329 PMC7003854

[B98] Wallace M. Bartz S. R. Chang W. L. Mackenzie D. A. Pauza C. D. Malkovsky M. (1996). Γ δ T lymphocyte responses to HIV. Clin. Exp. Immunol. 103, 177–184. 10.1046/j.1365-2249.1996.d01-625.x 8565297 PMC2200355

[B99] Wallace M. Gan Y. H. Pauza C. D. Malkovsky M. (1994). Antiviral activity of primate gamma delta T lymphocytes isolated by magnetic cell sorting. J. Med. Primatol. 23, 131–135. 10.1111/j.1600-0684.1994.tb00113.x 7966227

[B100] Wallace M. Scharko A. M. Pauza C. D. Fisch P. Imaoka K. Kawabata S.. (1997). Functional γ δ T-lymphocyte defect associated with human immunodeficiency virus infections. Mol Med. 3, 60–71. 10.1007/BF03401668 9132281 PMC2230098

[B101] Ward J. Bonaparte M. Sacks J. Guterman J. Fogli M. Mavilio D.. (2007). HIV modulates the expression of ligands important in triggering natural killer cell cytotoxic responses on infected primary T-cell blasts. Blood 110, 1207–1214. 10.1182/blood-2006-06-028175 17513617 PMC1939902

[B102] Wesch D. Hinz T. Kabelitz D. (1998). Analysis of the TCR Vγ repertoire in healthy donors and HIV-1-infected individuals. Int. Immunol. 10, 1067–1075. 10.1093/intimm/10.8.1067 9723692

[B103] Wilhelm M. Smetak M. Schaefer-Eckart K. Kimmel B. Birkmann J. Einsele H.. (2014). Successful adoptive transfer and *in vivo* expansion of haploidentical γδ T cells. J. Transl. Med. 12:45. 10.1186/1479-5876-12-45 24528541 PMC3926263

[B104] Willcox B. E. Willcox C. R. (2019). γδ TCR ligands: the quest to solve a 500-million-year-old mystery. Nat. Immunol. 20, 121–128. 10.1038/s41590-018-0304-y 30664765

[B105] Wong J. K. Yukl S. A. (2016). Tissue reservoirs of HIV. Curr. Opin. HIV AIDS 11, 362–370. 10.1097/COH.0000000000000293 27259045 PMC4928570

[B106] Wrobel P. Shojaei H. Schittek B. Gieseler F. Wollenberg B. Kalthoff H.. (2007). Lysis of a broad range of epithelial tumour cells by human γ δ T cells: involvement of NKG2D ligands and T-cell receptor- versus NKG2D-dependent recognition. Scand. J. Immunol. 66, 320–328. 10.1111/j.1365-3083.2007.01963.x 17635809

[B107] Wu D. Wu P. Wu X. Ye J. Wang Z. Zhao S.. (2015). *Ex vivo* expanded human circulating Vδ1 γδT cells exhibit favorable therapeutic potential for colon cancer. Oncoimmunology 4:e992749. 10.4161/2162402X.2014.992749 25949914 PMC4404819

[B108] Zhang T. He X. Tsang T. C. Harris D. T. (2004). Transgenic TCR expression: comparison of single chain with full-length receptor constructs for T-cell function. Cancer Gene. Ther. 11, 487–496. 10.1038/sj.cgt.7700703 15153936

